# A New Microarray Substrate for Ultra-Sensitive Genotyping of KRAS and BRAF Gene Variants in Colorectal Cancer

**DOI:** 10.1371/journal.pone.0059939

**Published:** 2013-03-25

**Authors:** Silvia Galbiati, Francesco Damin, Pamela Pinzani, Irene Mancini, Serena Vinci, Marcella Chiari, Claudio Orlando, Laura Cremonesi, Maurizio Ferrari

**Affiliations:** 1 San Raffaele Scientific Institute, Genomic Unit for the Diagnosis of Human Pathologies, Center for Translational Genomics and Bioinformatics, Milan, Italy; 2 Istituto di Chimica del Riconoscimento Molecolare, C. N. R., Milan, Italy; 3 Clinical Biochemistry Unit, Department of Clinical Physiopathology, University of Florence, Florence, Italy; 4 Vita-Salute San Raffaele University, Milan, Italy; 5 Diagnostica e Ricerca San Raffaele SpA, Milan, Italy; Baylor University Medical Center, United States of America

## Abstract

Molecular diagnostics of human cancers may increase accuracy in prognosis, facilitate the selection of the optimal therapeutic regimen, improve patient outcome, reduce costs of treatment and favour development of personalized approaches to patient care. Moreover sensitivity and specificity are fundamental characteristics of any diagnostic method. We developed a highly sensitive microarray for the detection of common KRAS and BRAF oncogenic mutations. In colorectal cancer, KRAS and BRAF mutations have been shown to identify a cluster of patients that does not respond to anti-EGFR therapies; the identification of these mutations is therefore clinically extremely important. To verify the technical characteristics of the microarray system for the correct identification of the KRAS mutational status at the two hotspot codons 12 and 13 and of the BRAF^V600E^ mutation in colorectal tumor, we selected 75 samples previously characterized by conventional and CO-amplification at Lower Denaturation temperature-PCR (COLD-PCR) followed by High Resolution Melting analysis and direct sequencing. Among these samples, 60 were collected during surgery and immediately steeped in RNAlater while the 15 remainders were formalin-fixed and paraffin-embedded (FFPE) tissues. The detection limit of the proposed method was different for the 7 KRAS mutations tested and for the V600E BRAF mutation. In particular, the microarray system has been able to detect a minimum of about 0.01% of mutated alleles in a background of wild-type DNA. A blind validation displayed complete concordance of results. The excellent agreement of the results showed that the new microarray substrate is highly specific in assigning the correct genotype without any enrichment strategy.

## Introduction

Defining the molecular signature of human cancers could be central to the development of a personalized approach to patient care. In fact the identification of appropriate biomarkers might increase accuracy in prognosis, facilitate the selection of the optimal therapeutic regimen, improve patient outcome, and reduce costs of treatment [1]. Thus, stratification of single patients based on molecular and genetic characteristics is the expected evolution of the modern clinical oncology.

Recently therapeutic agents targeting specific genetic variants and well characterized molecular pathways have been developed. This is the case of the oncogene KRAS which is part of the signaling pathway of several different molecules. Gain-of-function missense mutations are often somatically acquired in colorectal cancer, prevalently at three hot spots represented by codons 12, 13, and 61. Unfortunately, at these levels the number of substitutions is high, making their detection more complex with allele specific techniques. Constitutively, activating mutations at these hot spot sites can determine resistance to EGFR-targeted therapies which should otherwise significantly improve the survival rate and the quality of life of patients [2], [3], [4], [5]. The potential of KRAS codon 12/13 mutations as effective molecular markers for drug selection has received considerable attention leading to their use in the routine care of patients with colorectal cancer [6]. The European health authority (http://www.emea.europa.eu/pdfs/human/press/pr/27923508en.pdf.) as well as the American Society for Clinical Oncology [7] and the National Comprehensive Cancer Network (NCCN, http://www.nccn.org/professionals/physician_gls/PDF/colon.pdf.) require KRAS mutational analysis on colorectal cancer prior to anti-EGFR therapy.

Another promising biomarker of anti-EGFR resistance is represented by BRAF^V600E^ mutation, that occurs in about 10% of colorectal cancers. BRAF is the immediate downstream effector of KRAS in the Ras/Raf/MAPK signaling pathway and BRAF^V600E^ activating mutation is mutually exclusive for KRAS mutations [6]. Despite the currently limited data, and lack of complete consensus, it is likely that BRAF mutations have a role in determining the response to anti-EGFR mAb treatment and it is associated with worse prognosis, independently from treatment [8], [9]. Furthermore, patients with tumors carrying mutant BRAF might also benefit from selective BRAF inhibitors such as PLX-4032 [10].

In the present scenario of screening strategies, the current methods of analysis (conventional sequencing, pyrosequencing, etc.) are time-consuming, expensive and lack robustness. Another emerging issue is connected to the real sensitivity of these methods that seem to detect minority mutated alleles only when present at concentrations higher than 10–20%. In previous works [11], [12], we underlined the importance of sensitivity in the detection of minority mutated alleles in biological samples and confirmed the usefulness of COLD-PCR for their enrichment, particularly in samples with low percentages of tumour cells. On average, 15% of patients initially classified as negative for KRAS or BRAF^V600E^ variants were found positive after COLD-PCR [11], [12].

Microarrays represent an inexpensive and accurate tool for parallel genotyping of multiple markers, suitable for routine analysis in medical diagnostics [13]. Here, we report on the development of a highly sensitive microarray for the detection of KRAS and BRAF mutations. The microarray is developed using a crystalline silicon slide coated by a thermally grown silicon dioxide (SiO_2_) layer and functionalized by adsorption of a copolymer of dimethylacrylamide (DMA), N-acryloyloxysucinimide (NAS) and meta-acryloy propyl trimethoxy silane (MAPS), copoly(DMA-NAS-MAPS), originally developed for glass DNA microarrays [14]. The backbone of the polymer consists of DMA, a monomer that adsorbs to the surface by hydrogen bonding; NAS is the chemically reactive monomer that reacts covalently with bio-probes, whereas MAPS contribute to film stability by condensing with surface silanols. The coating procedure is simple and reproducible, when compared to organo-silanization, a process that requires highly controlled conditions and suffer from poor reproducibility. This functional polymer has been widely applied in the biosensor field for the bio-functionalization of polystyrene nanobeads [15], silicon microcantilevers [16], polydimethylsiloxane [17], and nitrocellulose [18] substrates.

The choice of silicon substrate coated by a layer of SiO_2_ of 100 nm thickness leads to a strong intensification of fluorescence on the surface resulting from optical constructive interference between the incident and reflected lights of the fluorescent radiation. The condition of constructive interference at the substrate surface is fulfilled in several types of glass slides coated with layers of dielectric or metal films [19]. However, the strategy involved in producing such complex multi-layer structures often suffers low reproducibility and difficult process control. The simplest configuration to achieve a fluorescent enhancement close to that provided by multi-layer slides consists of the silicon planar reflector coated with a thin film of SiO_2_ [20], [21] proposed by this work.

To evaluate technical performances of the newly developed platform and verify its ability to correct genotyping of KRAS and BRAF^V600E^ mutations in colorectal tumor samples, we selected 60 tissue samples already classified for these genetic variants by complete protocols of conventional and COLD-PCR, High Resolution Melting analysis and direct sequencing. The excellent outcome of the results will be discussed in order to show the advantages and the drawbacks of both technologies.

## Materials and Methods

### Ethics

Tissue samples were collected at Azienda Ospedaliera Universitaria Careggi in Florence (Italy) from 75 patients undergoing surgery for CRC in the period 1/6/2009–2/5/2011. All individuals enrolled in this study provided written informed consent. The study was approved by the review board of the University of Florence. Moreover, we used two cell lines provided by ATCC-LGC Standards Partnership: the CCRF-CEM cell line as reference for KRAS mutation p.G12D (heterozygous) and the human melanoma cell line A375 as the source of homozygous BRAF^V600E^ DNA. The human colorectal carcinoma cell line SW620 (used as reference for KRAS mutation pG12V, homozygousand the human breast cancer cell line MCF-7 (wild-type for both KRAS and BRAF genes) were supplied by Banca Biologica e Cell Factory (IRCCS Azienda Ospedaliera Universitaria San Martino - IST Istituto Nazionale per la Ricerca sul Cancro).

### Sample preparation

Sixty tissue samples were collected during surgery and immediately steeped in RNAlater (Qiagen Gmbh, Hilden, Germany) at 4°C for 24 hours and subsequently stored at −80°C until analysis. Tissues were disrupted by Tissue Lyser with Stainless Steel Beads 5 mm (Qiagen) according to the manufacturer's instructions. DNA purification was performed with QIAcube™ and QIAamp DNA mini Kit (Qiagen).

The concentration of DNA was determined with Nanodrop ND-1000 Spectrophotometer (Thermo Scientific, DE, USA).

Moreover, 15 FFPE tissues were also analyzed; DNA from FFPE tissues was extracted using the FFPE Tissue kit (Qiagen) following manufacturer's instruction.

DNA samples were firstly investigated by means of conventional PCR and COLD-PCR amplification followed by HRM and direct sequencing. Subsequently they were blindly submitted to the analysis by the newly developed microarray device to asses its ability in KRAS and BRAF mutations genotyping.

Positive control samples were represented by DNA of cell lines harboring mutations in the target genes (See previous section Ethics). In particular wild-type and mutant samples were assayed separately as single samples and as mixtures, in order to get known percentage of mutated allele (from 6% to 0.01%) to be used for the determination of assay sensitivity for KRAS p.G12D and BRAF V600E variants.

Moreover, plasmidic DNA containing the wild-type sequences and alternatively all the considered variants was used to obtain reconstituted samples to prove assay sensitivity and specificity for all the other KRAS mutations.

### Mutagenesis

Mutant-bearing plasmids were generated through the cloning of specific mutagenized PCR products harboring the seven mutations tested in the assay and the corresponding wild-type fragment. Mutagenized fragments were prepared using a modification of the method previously reported [22]. Briefly, mutagenesis was achieved by dividing each amplicon into two fragments. The 5′ fragment was then amplified with the original forward primer and a mutagenizing reverse primer introducing a conservative transversion. In parallel, the 3′ fragment was amplified with the original reverse primer and a mutagenized forward primer introducing the same nucleotide variation as above (see Table S1). This led to the production of two partially overlapping fragments, which were each gel-eluted in a final volume of 50–100 µL of distilled water to eliminate non-incorporated primers. We mixed 2 µL of each eluted solution together; each mixture was then elongated for 15 cycles in the presence of the PCR reaction mixture containing all reagents but primers. The product of elongation reaction, resulting in a full-length centrally mutagenized fragment, was further PCR amplified for 20–30 cycles by addition of the complete PCR mixture and cloned in the plasmid vector (TOPO TA Cloning, Invitrogen, LifeTechnologies, Milan, Italy) according to manufacturer's protocol. Direct sequencing confirmed that the desired nucleotide change was introduced into the mutagenized control.

### PCR conditions

Exon 2 of the KRAS gene was amplified with the following primer set: 5′- GCC TGC TGA AAA TGA CTG AA -3′ (forward) and 5′- AGA ATG GTC CTG CAC CAG TAA-3′ (5-amino-modified, reverse) generating a 167 bp fragment. Primers used for amplification of BRAF exon 15 (amplicon size 173 bp) were 5′- TGC TTG CTC TGA TAG GAA AAT G-3′ (forward) and 5′- CCA CAA AAT GGA TCC AGA CA-3′ (5-amino-modified, reverse).

PCR for both genes was performed in 25 µL reaction containing 15 ng of DNA, 200 uM of each deoxynucleotide, 10 mM Tris–HCl (pH 8.3), 50 m M KCl, 1.5 mM MgCl_2_, 1.5 U of DNA polymerase (AmpliTaq Gold; Applied Biosystems, Foster City, CA, USA) and 10 pmoles of each primer. Cycling conditions entailed an initial denaturation at 95°C for 10 min followed by 35 cycles at 95°C for 30 s, 58°C for 30 s and 72°C for 30 s, and a final elongation at 72°C for 10 min.

### PCR purification

After the amplification process, each amplicon was purified and desalted with use of a 96-well plate (Multiscreen-PCR Plates MANU 030) coupled with the Multiscreen Separation System (Millipore Corporation, Billerica, MA, USA). PCR products were eluted in 35 uL of 1× printing buffer (150 mM sodium phosphate pH 8.5).

### Reporter and stabilizer design

**Figure 1:**
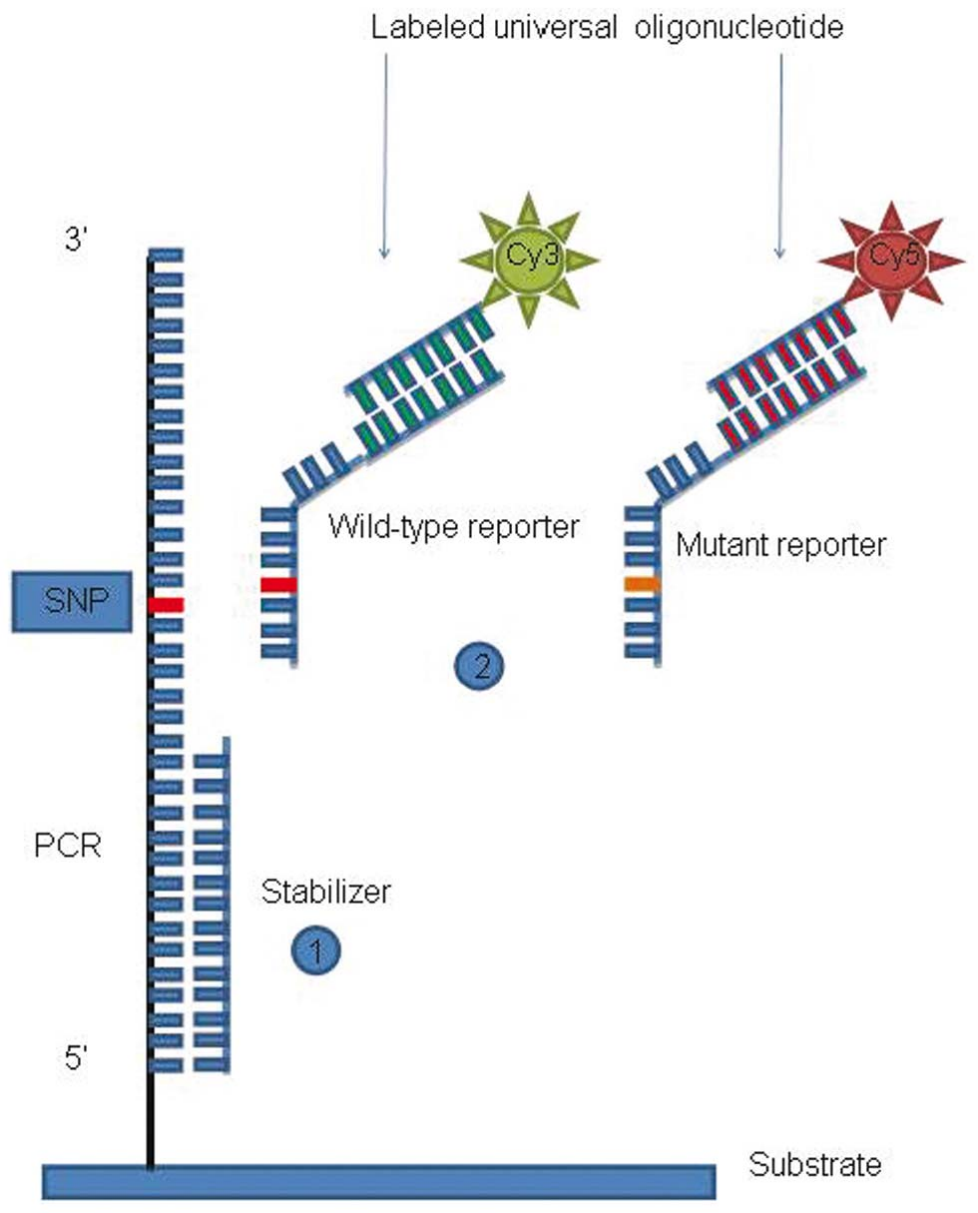
Assay scheme. Hybridization steps: 1) stabilizer oligonucleotide to open the secondary structure of the PCR fragment; 2) The “universal reporter mix”. The mix contains the wild-type and mutant reporters. Each reporter is prolonged by a tail complementary to the labeled universal oligonucleotide. Cyanine 3- (Cy3) and Cyanine 5- (Cy5) labeled universal oligonucleotide anneal to wild-type and mutant reporters, respectively.

Reporters were designed in a dot-blot format with the base variation corresponding to the hotspots located internally (Table 1). A universal labelling format was used (Figure 1), based on reporters containing a sequence-specific tail that hybridizes to universal Cy3 or Cy5 labelled probes (wild-type probe: CTC AAT GTT CGG ACT CAG-Cy3; mutant probe: TGT CAA GCG ATA TAC TGC-Cy5) [23].

**Table 1:** Sequences of reporters and stabilizer oligonucleotides.

Mutation(Aminoacid change)	Reporter sequences(Stabilizer sequences)	Hybridizationtemperature (°C)
**KRAS**		
Wild-type	5′-* ctgagtccgaacattgag-CTGGTGGCGTA-3′	
c.35G>C (p.G12A)	5′-† gcagtatatcgcttgaca-CTG**C**TGGCGTA-3′(5′-gcaagagtgccttgacgatacagctattcag-3′)	30
c.34G>T (p.G12C)	5′-† gcagtatatcgcttgaca- TGGAGCT**T**GTGG-3′(5′-gcaagagtgccttgacgatacagctattcag-3′)	41
c.35G>A (p.G12D)	5′-† gcagtatatcgcttgaca- GCTG**A**TGGCGT-3′(5′-gcaagagtgccttgacgatacagctattcag-3′)	37
c.34G>C (p.G12R)	5′-† gcagtatatcgcttgaca-CT**C**GTGGCGTA-3′(5′-gcaagagtgccttgacgatacagctattcag-3′)	30
c.34G>A (p.G12S)	5′-† gcagtatatcgcttgaca-GCT**A**GTGGCGTA-3′(5′-gcaagagtgccttgacgatacagctattcag-3′)	36
c.35G>T (p.G12V)	5′-† gcagtatatcgcttgaca-GCTG**T**TGGCG-3′(5′-gcaagagtgccttgacgatacagctattcag-3′)	29
c.38G>A (p.G13D)	5′-† gcagtatatcgcttgaca-TGGTG**A**CGTAGG-3′(5′-gcaagagtgccttgacgatacagctattcag-3′)	36
**BRAF**		
Wild-type	5′- * ctgagtccgaacattgag-GCTACAGTGAAATCT-3′	
V600E	5′- † gcagtatatcgcttgaca-GCTACAG**A**GAAATCT-3′Stab 1 (5′-cgatggagtgggtcccatcagtttgaa-3′)Stab 2 (5′-gaagacctcacagtaaaaataggtgatttt ggtcta-3′)	47

*† Universal reporter formats which hybridize to the universal probes (in small letter); * wild-type sequence-specific tail; ^†^ mutant sequence-specific tail.

Nucleotide numbering reflects cDNA numbering with +1 corresponding to the A of the ATG translation initiation codon in the reference GenBank sequence (NM_033360.2). The initiation codon is codon 1.

Stabilizer (oligonucleotide necessary to open the secondary structures present in the amplicon) and reporter design was performed with the help of web-free programs (DNAmfold server: http://www.bioinfo.rpi.edu/Ezukerm/rna and OligoAnalyzer 3.0 by Integrated DNA Technologies: http://www.idtdna.com) [24]. All reporters were designed using the antisense strand as target amplicon. All the 7 KRAS mutations were analyzed with the same stabilizer oligonucleotide reported in Table 1. The codon 600 BRAF mutation required the use of two stabilizers (Stab 1: located at 5′ to the mutation; Stab 2: located at 3′ to the mutation, reported in Table 1).

### Silicon slide coating and microarray preparation

Untreated silicon slides 1000A Thermal Oxide (14×14 mm^2^) were supplied by SVM, Silicon Valley Microelectronics Inc. (Santa Clara, CA USA). Silicon slides were pre-treated with 0.1 M sodium hydroxide for 30 min and washed with water and dried. After pre-treatment, silicon slides were immersed for 30 min in a copoly(DMA-NAS-MAPS) solution (1% w/v in 0.9 M (NH4)_2_SO_4_ water solution). Copoly(DMA-NAS-MAPS) was synthesized and characterized as described [14].

Slides were finally rinsed with water and dried under vacuum at 80°C.

The PCR products for each gene, amplified from primers with a 5′ primary amino-group, necessary to bind the amplicons covalently to the substrate through a reaction between the amino groups and the active esters of the polymer coating, were spotted on the microarray substrates. Three µL of the amino-modified amplicons were printed in 6 replicates using a piezoelectric spotter, SciFLEXARRAYER S5 (Scienion Germany), on coated silicon slides. An amino-modified oligonucleotide labelled with Cy3 was spotted as a positional reference in four columns in every array. Spotting conditions was carried out at as described [25]. The amplicons were coupled to the arrays by incubating in an uncovered storage box, laid in a sealed chamber, saturated with sodium chloride (40 g/100 mL H_2_O) and incubated at room temperature overnight.

After incubation, all residual reactive groups of the coating polymer were blocked by dipping the slide in pre-warmed blocking solution as reported [25].

### Hybridization

Immediately before hybridization, printed slides were dipped in 0.1 M NaOH for 5 min to denature the double-stranded immobilized amplicons, subsequently rinsed with water and dried. Sequences of reporters and stabilizers along with the hybridization temperatures are detailed in Table 1. In the first step, 0.5 µL of the stabilizer oligonucleotide were mixed with 49.5 µL of hybridization buffer (2× SSC, 0.1% SDS, 0.2 mg/mL BSA) up to 1 µM final concentration and spread onto the spotted area of the slide. The slides were incubated at 20°C for 30 min in the Thermomixer Comfort (Eppendorf) hybridization chamber, and then washed at room temperature in a 4× SSC buffer to remove the cover slip. This first wash step was followed by a brief wash (30 s) in a low-salt buffer (0.2× SSC). Then, for the detection of G12S, G12D, G12C, G12R, G13D KRAS mutations and for the BRAF^V600E^ mutations, the reporter for the wild-type and the mutated sequences and their corresponding universal oligonucleotides labelled with Cy3 and Cy5 respectively, were mixed together in equimolar amounts (final concentration 1 µM) and added to the hybridization buffer (2× SSC, 0.1% SDS, 0.2 mg/mL BSA) (see Figure 1 for the assay scheme). In the case of G12A and G12V KRAS mutations it was necessary to incubate the amplicons in two consecutive steps. In the first step the amplicons were hybridized with the reporter complementary to the mutated sequence together with the corresponding universal oligonucleotide labeled with Cy5; then, after a brief wash in 4× SSC to remove the cover slip, in the second one the amplicons were hybridized with the reporter complementary to the wild-type and its corresponding universal oligonucleotide labeled with Cy3. Both incubations lasted for 1 hour.

Finally the silicon slides were removed from the hybridization chamber and soaked briefly in 4×SSC buffer to remove the cover slip, washed twice for 5 min in 2× SSC/0.1% SDS, pre-warmed at the specific hybridization temperature, then dipped, in sequence, in a solution 0.2× SSC and 0.1× SSC for 1 min at room temperature, dried by centrifuging at 780 rpm for 3 min and scanned.

### Image scanning and data analysis

ProScanArray (Perkin Elmer, MA, USA) was used to scan the hybridized slides. In particular a green laser (λ_ex_ 543 nm/λ_em_ 570 nm) for the Cy3 dye and a red laser (λ_ex_ 633 nm/λ_em_ 670 nm) for the Cy5 dye were applied. The photomultiplier (PMT) tube gain and the laser power changed between fluorochromes and different experiments. 16-bit TIFF images were analyzed at 5 µm resolution. Data intensities were extracted with the scanner software and the data analysis was performed for each experiment as previously described [25].

### Conventional and COLD-PCR amplification followed by High Resolution Melting (HRM) and direct sequencing

Conventional PCR, COLD-PCR, HRM and sequencing protocols have been already described [11], [26].

## Results

### 1) Conventional and COLD-PCR amplification HRM and sequencing results

Initially, all of the 75 samples were screened for the research of KRAS mutations and BRAF^V600E^ by HRM and sequencing. In a second phase, all samples (mutated and wild-type) were resubmitted to a complete screening using COLD-PCR amplification followed by HRM and sequencing as already reported [11].

Globally, 59 out of 75 samples contained alternatively one mutation either on KRAS hotspots or the BRAF^V600E^ variant, while 16 were classified as wild type samples and included as negative controls. It is important to note that in 9/59 mutated samples, the identification of base substitutions was only made possible through the use of fast or full COLD-PCR protocols, probably due to the lower percentages of mutated alleles in the starting samples. These samples were appositely introduced in the validation study to test the sensitivity of the proposed platform. For a detailed description of the distribution of KRAS and BRAF variants in our samples see Table 2.

**Table 2:** Molecular features of all patients enrolled by HRM-direct sequencing, and microarray.

Sample Number	HRM-Sequencing	Array	Sample Number	HRM-Sequencing	Array
1	p.G12D	p.G12D	31	p.G13C *	wt *
2	V600E	V600E	32	p.G12V	p.G12V
3	p.G12S	p.G12S	33	wt	wt
4	p.G12V	p.G12V	34	p.G12R	p.G12R
5	p.G12V	p.G12V	35	wt	wt
6	p.G12D	p.G12D	36	p.G12V	p.G12V
7	p.G13D	p.G13D	37	p.G12D	p.G12D
8	p.G12D	p.G12D	38	wt	wt
9	p.G12D	p.G12D	39	wt	wt
10	p.G12D†	p.G12D	40	p.V14I *	wt *
11	p.G12C	p.G12C	41	p.G12V†	p.G12V
12	p.G13D	p.G13D	42	p.G13D†	p.G13D
13	p.G12D†	p.G12D	43	p.G12V†	p.G12V
14	p.G12A	p.G12A	44	p.G12V	p.G12V
15	p.G13D†	p.G13D	45	p.G12D	p.G12D
16	p.G12A†	p.G12A	46	wt	wt
17	p.G12S	p.G12S	47	V600E	V600E
18	p.G13D	p.G13D	48	wt	wt
19	V600E	V600E	49	wt	wt
20	p.G12C	p.G12C	50	wt	wt
21	V600E	V600E	51	wt	wt
22	V600E	V600E	52	p.G12D	p.G12D
23	p.G12D	p.G12D	53	wt	wt
24	p.G12V	p.G12V	54	p.G12C	p.G12C
25	p.G12A	p.G12A	55	V600E	V600E
26	p.G12V†	p.G12V	56	wt	wt
27	p.G13D	p.G13D	57	p.G12V	p.G12V
28	V600E	V600E	58	p.G12C	p.G12C
29	p.G12V	p.G12V	59	p.G13D	p.G13D
30	p.G12C†	p.G12C	60	p.G12A	p.G12A
1 FFPE	p.G12A	p.G12A	2 FFPE	V600E	V600E
3 FFPE	wt	wt	4 FFPE	p.G12R	p.G12R
5 FFPE	wt	wt	6 FFPE	wt	wt
7 FFPE	wt	wt	8 FFPE	p.G13D	p.G13D
9 FFPE	wt	wt	10 FFPE	V600E	V600E
11 FFPE	p.G12C	p.G12C	12 FFPE	p.G12C	p.G12C
13 FFPE	V600E	V600E	14 FFPE	V600E	V600E
15 FFPE	V600E	V600E			

* mutation detected only by HRM and sequencing; by microarray analysis, the sample was identified as wild-type since no specific reporter was designed for the mutation.

† mutation detected by COLD-PCR and sequencing, non detected by conventional PCR.

### 2) Optimization of silicon slide analysis

**Figure 2:**
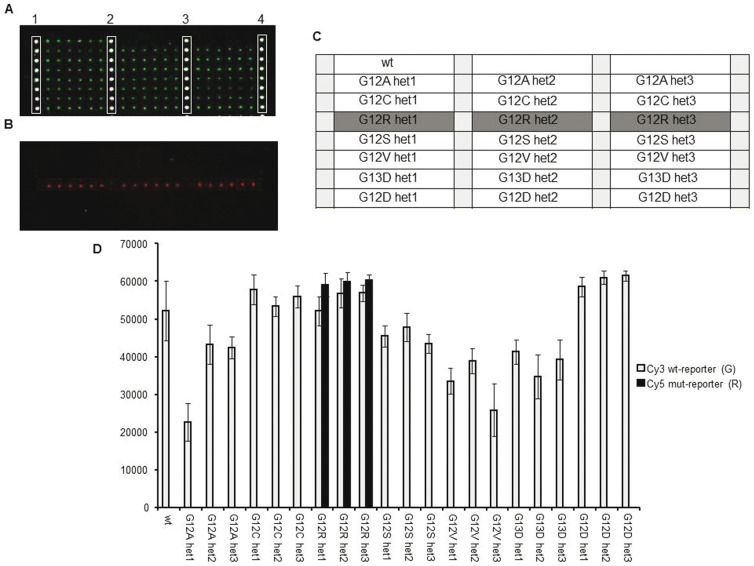
Microarray image for genotyping the G12R KRAS mutation. (A) microarray scanning of the Cy3 fluorescence signal corresponding to the wild-type allele. Spots in column 1,2,3,4 represent amino-modified oligonucleotide labelled with Cy3 used as reference spots. (B) scanning of the Cy5 fluorescence signal corresponding to the mutated allele. (C) microarray spotting scheme. wt: wild-type control samples; het1, het2 and het3: heterozygous control samples for G12A, G12C, G12R, G12S, G12V, G13D; G12D KRAS mutations; light grey squares represent amino-modified oligonucleotide labelled with Cy3 used as reference spots. (D) normalized relative fluorescence intensity after hybridization of known control samples with the reporters complementary to the G12R variation. Bars are the average of the intensity of the 6 replicates of each sample. The error bars are the standard deviations of the fluorescence intensity of each sample.

**Figure 3:**
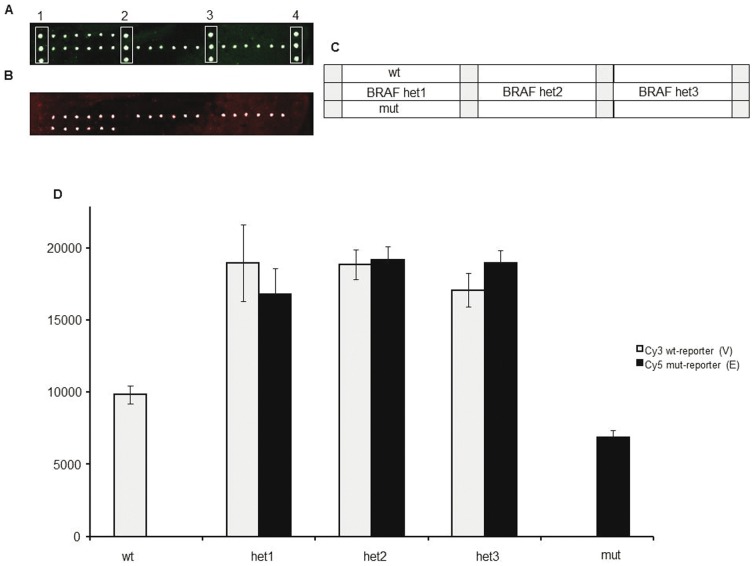
Microarray image for genotyping the V600E BRAF mutation. (A) Cy3 fluorescence signal corresponding to the wild-type allele. Spots in column 1,2,3,4 represent amino-modified oligonucleotide labelled with Cy3 used as reference spots. (B) Cy5 fluorescence signal corresponding to the mutated allele. (C) microarray spotting scheme. wt: wild-type control samples; het1, het2 and het3: heterozygous control samples; mut: homozygous mutant control sample; light grey squares represent amino-modified oligonucleotide labelled with Cy3 used as reference spots. (D) normalized relative fluorescence intensity after hybridization of known control samples with the reporters complementary to the V600E BRAF variation. Bars are the average of the intensity of the 6 replicates of each sample. The error bars are the standard deviations of the fluorescence intensity of each sample.

Wild-type control samples and reconstituted heterozygous control samples (generated by properly mixing plasmidic DNA containing wild-type and mutated sequences of all 7 KRAS mutations and of the BRAF^V600E^ variant) were used to test the assay specificity. Following optimization of temperature and hybridization time, a correct identification of all genotypes was achieved. An example is illustrated in Figures 2 and 3 where a hybridization experiment for the G12R KRAS and the BRAF^V600E^ mutation is shown, respectively. In the optimized system, for all the analyzed mutations we could evidence that: i) fluorescent signals were high, ii) no cross-hybridization was obtained, even for variants that affect the same or adjacent nucleotide positions (Figure 2D) and iii) a good reproducibility from spot to spot (shown by error bars) was found.

### 3) Sensitivity of the assay

Sensitivity of the assay in discriminating different proportions of mutated alleles was evaluated on serial dilutions (6%, 3%, 1,6%, 0,8%, 0.4%, 0.2%, 0.1%, 0.05%, 0.02%, 0.01% mutated/wild-type) of mutated DNA opportunely mixed with wild-type DNA. In particular we used CCRF-CEM cell line as reference for KRAS mutation p.G12D (heterozygous), the human colorectal carcinoma cell line SW620 (used as reference for KRAS mutation pG12V, homozygous), and the human melanoma cell lines A375 (used as the source of homozygous BRAF ^V600E^ DNA). The human breast cancer cell line MCF-7 were used as wild-type for both KRAS and BRAF genes. For all the other KRAS mutations we used the mutant-bearing plasmids generated.

**Figure 4:**
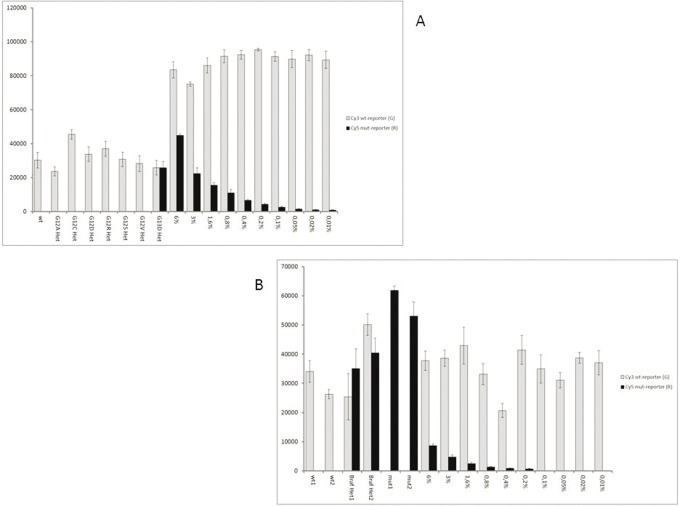
Relative fluorescence intensity for detecting the sensitivity of the system. Detection limit of the G13D mutation (A) and of the V600E BRAF mutation (B). wt: wild-type control samples; het1, het2 and het3: heterozygous control samples; numbers from 1 to 10: serial dilutions, 1 = 6%, 2 = 3%, 3 = 1,6%, 4 = 0,8%, 5 = 0.4%, 6 = 0.2%, 7 = 0.1%, 8 = 0.05%, 9 = 0.02%, 10 = 0.01% respectively. Bars are the average of the intensity of the 6 replicates of each sample. The error bars are the standard deviations of the fluorescence intensity of each sample.

The detection limit of the proposed method was different for the 7 KRAS mutations tested and for the V600E BRAF mutation. In particular, the microarray system has been able to detect a minimum of about 0.01% of mutated alleles in a background of wild-type DNA for the G13D mutation, about 0.025% of mutated allele for the G12R mutation, 0.05% for the G12C mutation, 0.1% for the G12D mutation, 0.2% for the V600E BRAF mutation, 0.4% for the G12A and the G12S mutations and 0.8% for the G12V respectively. In Figure 4 two examples of the results obtained for the G13D KRAS mutation and for the BRAF ^V600E^ mutation are shown.

### 4) Blind validation

**Figure 5:**
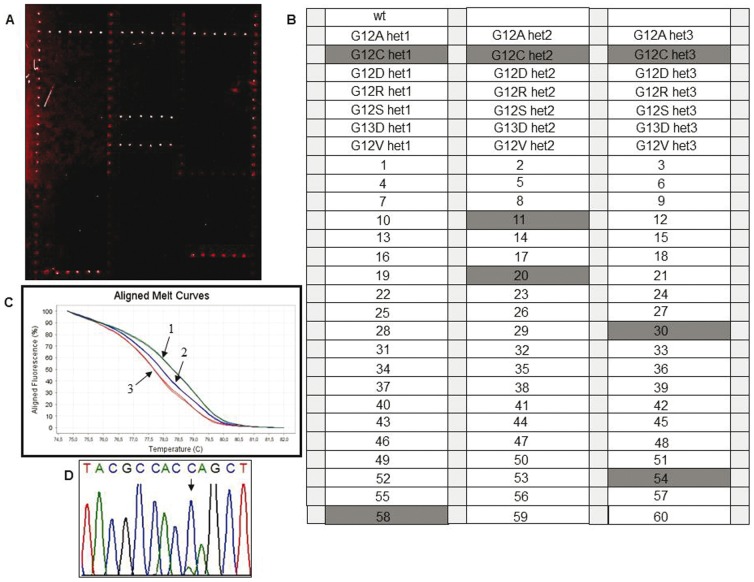
Microarray image for the analysis of the G12C KRAS mutation samples. (A) Cy5 fluorescence signal corresponding to the mutated allele. (B) spotting scheme. wt: wild-type control samples; het1, het2 and het3: heterozygous control samples for G12A, G12C, G12D, G12R, G12S, G13D, G12V KRAS mutations; numbers from 1 to 60: sixty different solid tumour DNA samples. Grey markers represent the samples positive for G12C mutation; light grey squares represent an amino-modified oligonucleotide labelled with Cy3 used as reference spots. (C) HRM analysis of sample number 30 (number 2) compared to melting profiles of control samples (wild-type reference = number 1; mutated reference = number 3) after amplification by COLD-PCR: the melting behaviour is suggestive for the presence of mutated DNA in the sample. (D) direct sequencing analysis of sample number 30 submitted to COLD-PCR amplification protocol: the electropherogram confirms the presence of mutated DNA (G12C) in the sample.

**Figure 6:**
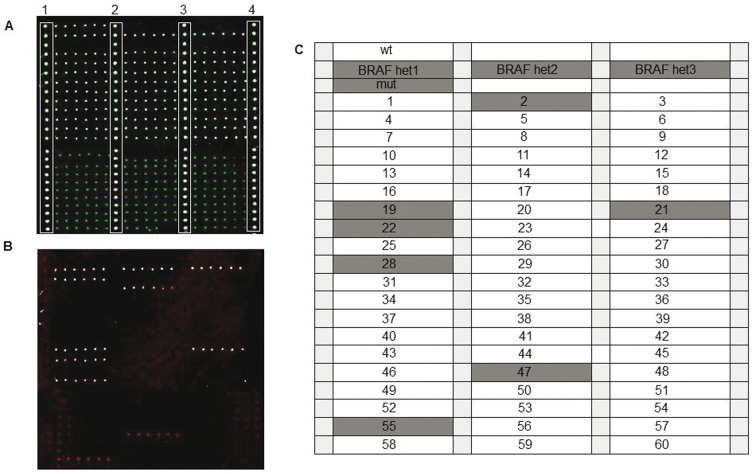
Microarray image for the analysis of the V600E BRAF mutation samples. (A) Cy3 fluorescence signal corresponding to the wild-type allele. Spots in column 1,2,3,4 represent amino-modified oligonucleotide labelled with Cy3 used as reference spots. (B) Cy5 fluorescence signal corresponding to the mutated allele. (C) spotting scheme. wt: wild-type control samples; BRAF het1, het2 and het3: heterozygous control samples; mut: homozygous mutant control sample; numbers from 1 to 60: sixty different solid tumour samples. Grey markers represent the samples positive for BRAF mutation; light grey squares represent an amino-modified oligonucleotide labelled with Cy3 used as reference spots.

**Figure 7:**
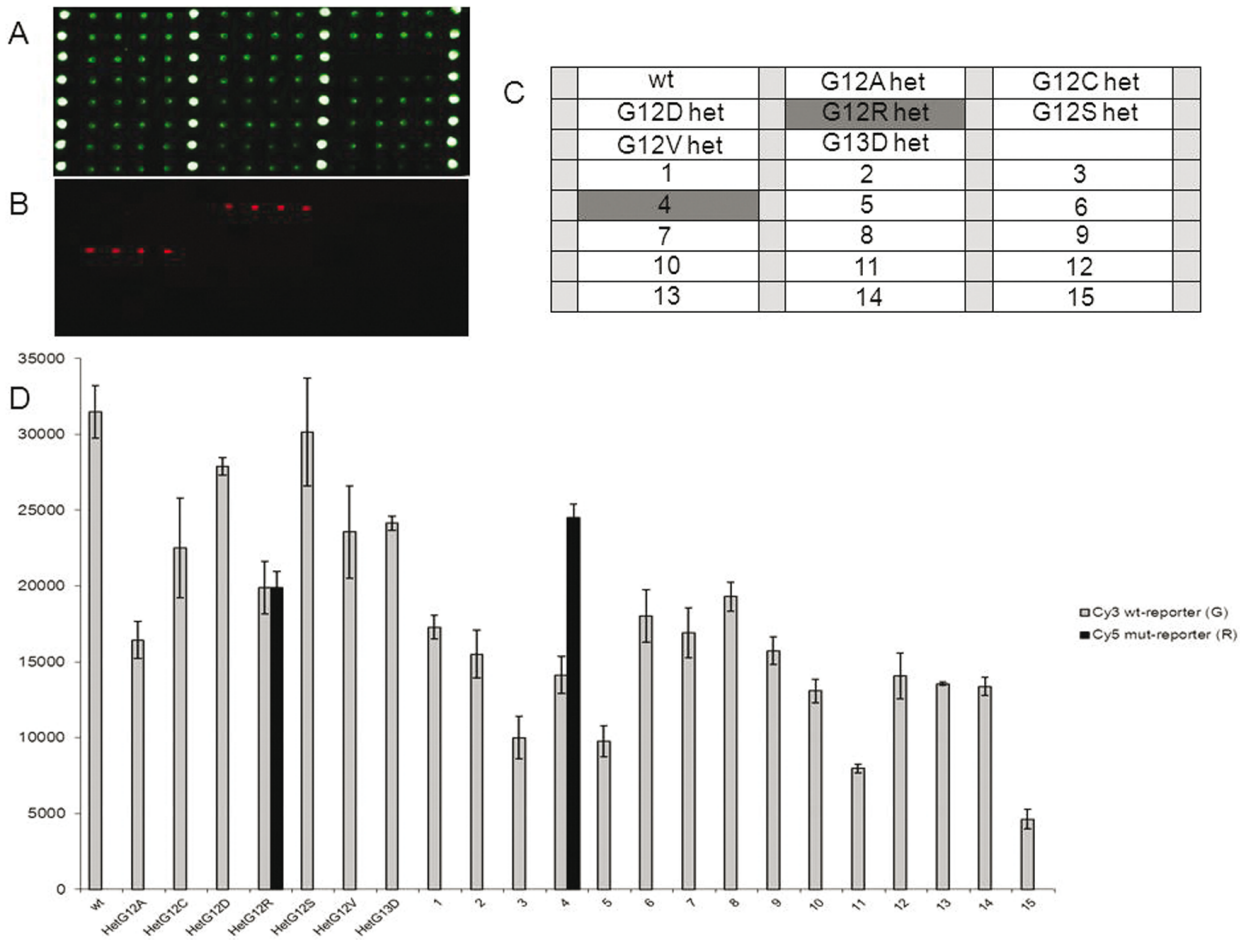
Microarray image for the analysis of the G12R KRAS mutation in FFPE samples. (A) Cy3 fluorescence signal corresponding to the wild-type allele. Spots in column 1,2,3,4 represent amino-modified oligonucleotide labelled with Cy3 used as reference spots. (B) Cy5 fluorescence signal corresponding to the mutated allele. (C) spotting scheme. wt: wild-type control samples; het: heterozygous control samples for G12A, G12C, G12D, G12R, G12S, G12V, G13D, KRAS mutations; numbers from 1 to 15: fifteen different FFPE solid tumour DNA samples. Grey markers represent the samples positive for G12R mutation; light grey squares represent an amino-modified oligonucleotide labelled with Cy3 used as reference spots. (D) Relative fluorescence intensity for detecting the sensitivity of the system evaluated for the G12R mutation. wt: wild-type control samples; het: heterozygous control samples; numbers from 1 to 15: FFPE samples. Bars are the average of the intensity of the 6 replicates of each sample. The error bars are the standard deviations of the fluorescence intensity of each sample.

**Figure 8:**
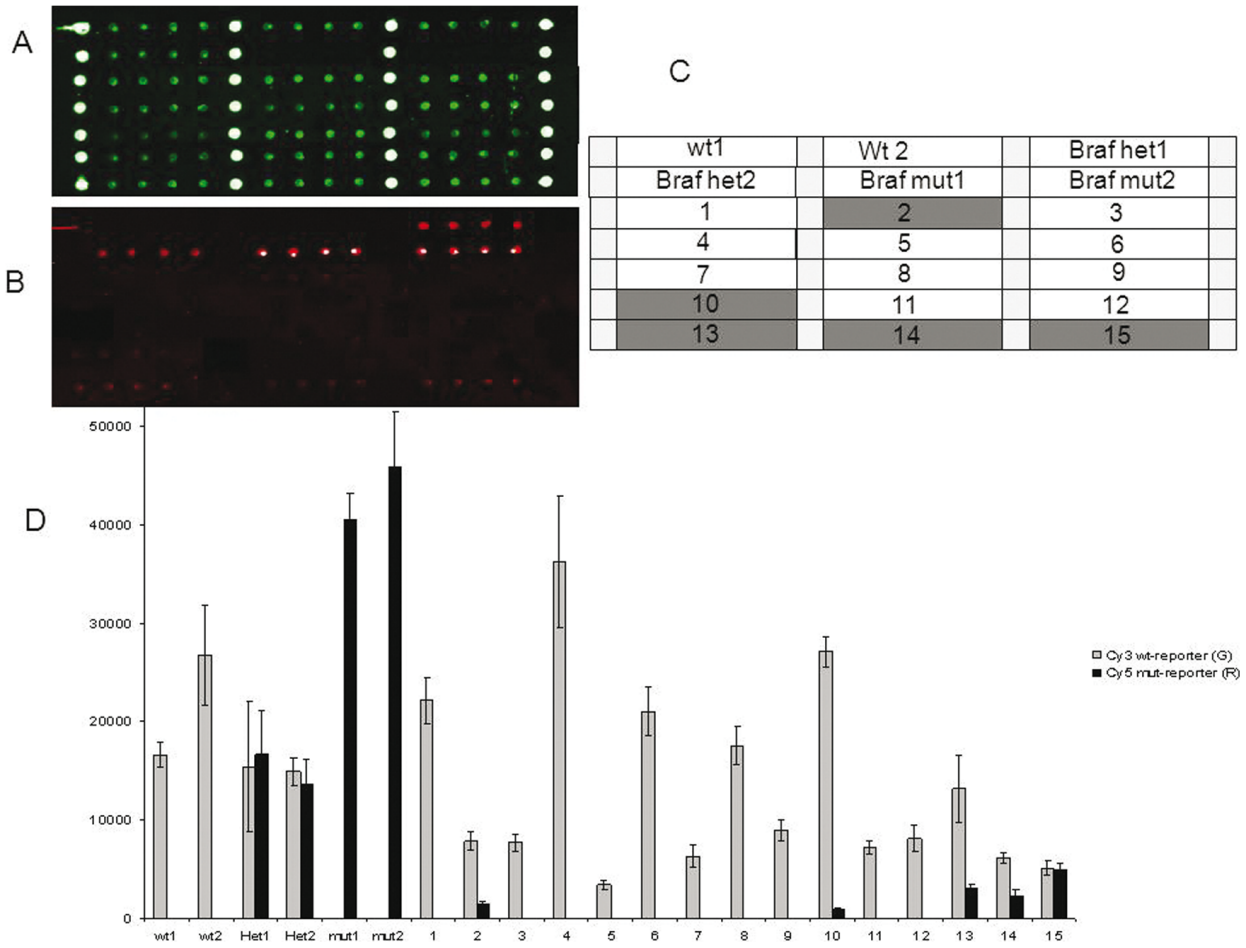
Microarray image for the analysis of the V600E BRAF mutation in FFPE samples. (A) Cy3 fluorescence signal corresponding to the wild-type allele. Spots in column 1,2,3,4 represent amino-modified oligonucleotide labelled with Cy3 used as reference spots. (B) Cy5 fluorescence signal corresponding to the mutated allele. (C) spotting scheme. wt: wild-type control samples; BRAF het1, het2: heterozygous control samples; BRAF mut1, mut2: homozygous mutant control sample; numbers from 1 to 15: fifteen different FFPE solid tumour DNA samples. Grey markers represent the samples positive for BRAF mutation; light grey squares represent an amino-modified oligonucleotide labelled with Cy3 used as reference spots. (D) Relative fluorescence intensity for detecting the sensitivity of the system evaluated for the V600E BRAF mutation. wt: wild-type control samples; het: heterozygous control samples; numbers from 1 to 15: FFPE samples. Bars are the average of the intensity of the 6 replicates of each sample. The error bars are the standard deviations of the fluorescence intensity of each sample.

Assay validation was performed in a blinded fashion by analyzing 75 samples from subjects either positive or wild-type for KRAS and BRAF mutations, previously characterized by HRM and direct sequencing. As an example, the microarray results for the G12C KRAS mutation and for BRAF^V600E^ mutation for frozen tissues are shown in Figures 5 and 6, respectively. Moreover in Figure 7 and 8 the microarray results for the G12R KRAS mutation and for BRAF^V600E^ mutation in FFPE samples are shown, respectively. The system was highly specific in assigning the correct genotype to all samples. Blind validation displayed complete concordance of results (Table 2) with the expected exception of samples N.31 and N.40, carrying the p.G13C and the p.V14I KRAS variants, which were expressly introduced to test the specificity of the newly developed probes (see Table 2).

## Discussion

Sensitivity and specificity are fundamental characteristics of any diagnostic method. Highly sensitive assays, able to detect small quantities of the substance under investigation, may open new opportunities in several clinical applications. In particular, the identification of minority mutated alleles within an excess of wild type alleles that represents a common problem in the analysis of cancer DNA, is technically very challenging, and requires the use of highly sensitive techniques.

Within this context, we aimed at developing innovative methodologies to improve the detection of very low proportions of cancer mutations. In this work, rapid detection of KRAS and BRAF mutations was achieved using a microarray support based on high-sensitivity silicon slides. Nevertheless, the availability of a slide with optimized optical properties is just one step toward the improvement of assay sensitivity. In order to fully take advantage of its characteristics, the surface must have high binding capacity and high hybridization yield of the immobilized molecules with their targets in solution. Hence, it has to be pointed out that the approach followed for the functionalization of the slides to allow the immobilization of the amino-modified amplicons is crucial for the success of the assay. Thus, taking into account these requirements, we have employed, for amplicon immobilization on silicon oxide surfaces, a coating procedure that our group has introduced to functionalize glass, silicon, and other kind of slides [27]. The combination of surface chemistry and optical slides properties leads to strong sensitivity increase with an enhancement of fluorescence signals from four to six times [20] with respect to glass. The low non-specific background provided by the polymer coating is an essential requirement to take advantage of fluorescence intensification as the signal enhancement is not specific for spot fluorescence. Moreover, the coating procedure is simple and reproducible and on the surface the polymer forms a thin film of few nanometers the morphology of which does not alter the optical properties of Si/SiO_2_ substrates.

By applying this system, our data indicated the possibility of identifying at least 0.01% of mutated alleles in a background of wild-type DNA, based on a dilution curve obtained by serial dilutions of mutated cancer cell lines and wild-type DNA. This theoretical sensitivity seems well suited for detecting even a limited percentage of mutated alleles in a heterogeneous sample, as obtained from CRC random tissue biopsies. Notably, our previous experience based on the comparison of conventional PCR versus COLD-PCR amplification [11] indicates that HRM and direct sequencing can identify up to 6.2% and 3.1% of mutated allele if preceded respectively by conventional and COLD-PCR amplification [12]. It is relevant to notice how just the use of COLD-PCR amplification could be determinant for the identification of the presence of BRAF^V600E^ or KRAS mutations at levels below the sensitivity of conventional methods [11], [12]. Nevertheless, other qPCR assays recently introduced on the market (TheraScreen®: K-RAS Mutation Kit, Elucigene KRAS.BRAF and Exigon) declare sensitivity levels of 1%, that represent a definite improvement compared to conventional PCR and sequencing methods, but they might be less sensitive that the method presented in the paper. Our approach could allow for genetic variants detection down to the level of 0.8–0.01% mutant allele (depending on each mutation), which is approximately 10–100 fold more sensitive than most of the other available assays.

To test the assay specificity, the system was initially validated on artificial heterozygous control samples containing wild-type and mutated sequences of all 7 KRAS mutations and BRAF^V600E^ mutation. The optimized slide system provided high fluorescence signals, good reproducibility and allowed correct identification of all genotypes. The drawbacks of this system are that the hybridization temperature must be carefully optimized for each mutation to obtain a specific assignment of the genotype and that the hybridization temperature is different for each mutation requiring 7 different microarrays for the 7 KRAS mutations. Nevertheless the great advantage of the array is that on the same slide you can spot several samples collected from different patients; it means that it is possible to screen disease-related mutations of up to one hundred of individuals on one slide. In this way, hundreds of genomic samples can be scored in a single experiment, making it particularly useful for screening large populations for important markers, such as those implicated in disease susceptibility.

Subsequent blind validation on 75 previously characterized samples from subjects either positive or wild-type for KRAS and BRAF mutations indicated 100% agreement for both FFPE and frozen tissue. The microarray's performance obtained in the analysis of FFPE samples is the same shown for the analysis of DNA extracted from tissue immerse in RNA later where the DNA integrity is excellent. This condition makes the array useful also in a clinical setting where the available tumour samples are often FFPE tissues.

All these data strongly support the application of the newly developed microarray assay for the scanning of unknown DNA samples.

KRAS and BRAF mutations are mutually exclusive. In the current study all 45 KRAS positive samples were wild-type for BRAF^V600E^ mutation and all 12 BRAF positive samples were wild-type for KRAS, supporting the mutual exclusiveness of the two genes in the tumour process.

The development of target therapies has created a clinical need for a fast and accurate molecular characterisation of tumours.

Understanding which gene is involved in the cancer origin is mandatory for good clinical practice and allows the physician to offer their patients better therapeutic choices.

Finally, the method described in this paper represents the first attempt to generate a platform for the simultaneous and sensitive detection of a panel of somatic mutations starting from a limited DNA amount. Obviously, this prototypical approach can be implemented to reduce cumbersome procedures and introduce semi-automatic steps.

Moreover, sensitivity is not to be considered a secondary aspect in view of a possible application to detect mutated alleles in biofluids, i.e. blood from cancer patients.

## Supporting Information

Table S1 **Gene variant, primer sequences, and base substitution for PCR-mediated site-directed mutagenesis.**
(DOC)
