# Supplementary material for: A New Microarray Substrate for Ultra-Sensitive Genotyping of KRAS and BRAF Gene Variants in Colorectal Cancer
Source: PLoS One. 2013 Mar 25;8(3):e59939. doi: 10.1371/journal.pone.0059939 (PMC3607556; doi:10.1371/journal.pone.0059939)
Supplement: Table S1 — Gene variant, primer sequences, and base substitution for PCR-mediated site-directed mutagenesis. (DOC) [file pone.0059939.s001.doc]

Table S1. Gene variant, primer sequences, and base substitution for PCR-mediated site-directed mutagenesis.

| MUTATION | MUTAGENESIS | MUTAGENIZED PRIMERS |
| --- | --- | --- |
| KRAS p.G12D | G>A | **F** 5'-GGA GCT G**A**T GGC GTA GCC-3' |
|  |  | **R** 5'-GGC TAC GCC A**T**C AGC TCC-3' |
| KRAS p.G12V | G>T | **F** 5'-GGA GCT G**T**T GGC GTA GCC-3' |
|  |  | **R** 5'-GGC TAC GCC A**A**C AGC TCC-3' |
| KRAS p.G12A | G>C | **F** 5'-GGA GCT G**C**T GGC GTA GCC-3' |
|  |  | **R** 5'-GGC TAC GCC A**G**C AGC TCC-3' |
| KRAS p.G12C | G>T | **F** 5'-GGA GCT **T**GT GGC GTA GCC-3' |
|  |  | **R** 5'-GGC TAC GCC AC**A** AGC TCC-3' |
| KRAS p.G12S | G>A | **F** 5'-GGA GCT **A**GT GGC GTA GCC-3' |
|  |  | **R** 5'-GGC TAC GCC AC**T** AGC TCC-3' |
| KRAS p.G12R | G>C | **F** 5'-GGA GCT **C**GT GGC GTA GCC-3' |
|  |  | **R** 5'-GGC TAC GCC AC**G** AGC TCC-3' |
| KRAS p.G13D | G>C | **F** 5'-GGA GCT GGT G**A**C GTA GCC-3' |
|  |  | **R** 5'-GGC TAC G**T**C ACC AGC TCC-3' |
| BRAF p.V600E | T>A | **F** 5'-TAG CTA CAG **A**GA AAT CTC-3' |
|  |  | **R** 5'-GAG ATT TC**T** CTG TAG CTA-3' |
